# Supplementary material for: Empirical methods for controlling false positives and estimating confidence in ChIP-Seq peaks
Source: BMC Bioinformatics. 2008 Dec 5;9:523. doi: 10.1186/1471-2105-9-523 (PMC2628906; doi:10.1186/1471-2105-9-523)
Supplement: Additional File 1 — A variety of html documents from the USeq web site detailing the available applications, their best usage, output file type descriptions, command line menus, etc. [file 1471-2105-9-523-S1.zip › USeqUserGuides/JavaDoc.zip!/JavaDoc/index-files/index-4.html]

D-Index


|  |  |  |
| --- | --- | --- |
| **Overview** \| Package \| Class \| Use \| **Tree** \| **Deprecated** \| **Index** \| **Help** | |  |
| **PREV LETTER**   **NEXT LETTER** | **FRAMES**    **NO FRAMES**     **All Classes** |  |


A B C D E F G H I J L M N O P Q R S T U V W X Z 

---


## **D**

**databaseDriver** - Static variable in class cgar.Util: **databaseName** - Static variable in class cgar.Util: **databasePassword** - Static variable in class cgar.Util: **databaseURL** - Static variable in class cgar.Util: **databaseUser** - Static variable in class cgar.Util: **DataLine** - Class in edu.utah.seq.methylation454: **DataLine(String)** - Constructor for class edu.utah.seq.methylation454.DataLine: **DBUtil** - Class in bioroot: Utility class to hold database relevant methods and fields, some from Hall and Brown The following is a collection of stuff for interacting with a SQL database First open a connection by calling connectMySQL, then get results with executeSQL, then close the connection with closeConnection. **DBUtil()** - Constructor for class bioroot.DBUtil: **DBUtil(String)** - Constructor for class bioroot.DBUtil: **decompressIt(File)** - Method in class edu.utah.seq.compression.CompressFasta: **defaults** - Variable in class util.apps.JQSub: **DefinedRegionScanSeqs** - Class in edu.utah.seq.analysis: Takes PointData and a set of defined regions, either a UCSC gene table or a bed file and scores the regions. **DefinedRegionScanSeqs(String[])** - Constructor for class edu.utah.seq.analysis.DefinedRegionScanSeqs: **delete(String[])** - Method in class util.amazon.S3Manager: Attempts to delete keyed objects from S3. **delete(String)** - Method in class util.amazon.S3Manager: Attempts to delete keyed object from S3. **deleteAntibody(AntibodyBean, UserBean, DBUtil)** - Static method in class bioroot.antibody.AntibodyModify: Attempts to delete an antibody, if sucessful, returns a "" String, otherwise an error message. **deleteAntibodyPrepIndex(AntibodyBean, DBUtil, HashMap)** - Static method in class bioroot.antibody.AntibodyBase: Scans a hashmap for an antibody prep to delete returns the index number **deleteDirectory(File)** - Static method in class util.gen.IO: Deletes a directories contents, and then the directory. **deleteFile(String)** - Static method in class gata.main.GATAUtil: **deleteFile(File)** - Static method in class util.gen.IO: Attempts to delete a file. **deleteFile(String)** - Static method in class util.gen.IO: Attempts to delete a file. **deleteFiles(File, String)** - Static method in class util.gen.IO: Deletes files in a given directory with a given extension. **deleteFiles(File, String, int)** - Static method in class util.gen.IO: Deletes files in a given directory with a given prefix, that are older cutoff. **deleteFiles(File[])** - Static method in class util.gen.IO: Attempts to delete the array of File. **deleteFiles(String, String)** - Static method in class util.gen.IO: Looks in the given directory for files ending with given String and deletes them. **deleteFilesNotEndingInExtension(File, String)** - Static method in class util.gen.IO: Deletes files in a given directory that don't end in a given extension. **deleteIntersectingIntervals()** - Method in class trans.main.IntervalFilter: **DeleteMeNow** - Class in bioroot: **DeleteMeNow()** - Constructor for class bioroot.DeleteMeNow: **deleteOligo(OligoBean, UserBean, DBUtil)** - Static method in class bioroot.oligo.OligoModify: Attempts to delete an oligo, if sucessful, returns a "" String, otherwise an error message. **deletePlasmid(PlasmidBean, UserBean, DBUtil)** - Static method in class bioroot.plasmid.PlasmidModify: Attempts to delete an plasmid, if sucessful, returns a "" String, otherwise an error message. **deleteSpecificIntervals()** - Method in class trans.main.IntervalFilter: **deleteSplitNormalizedFiles()** - Method in class trans.main.T2: **deleteStrain(StrainBean, UserBean, DBUtil)** - Static method in class bioroot.strain.StrainModify: Attempts to delete an strain, if sucessful, returns a "" String, otherwise an error message. **deleteTempFiles()** - Method in class util.bio.wrappers.Segment: **deleteUseDilutionIndex(AntibodyBean, DBUtil, HashMap)** - Static method in class bioroot.antibody.AntibodyBase: Scans a hashmap for a use dilution to delete returns the index string or null. **Delme** - Class in trans.misc: **Delme()** - Constructor for class trans.misc.Delme: **Delme2** - Class in util.apps: For testing **Delme2()** - Constructor for class util.apps.Delme2: **DelmeAligner** - Class in gata.aligner: **DelmeAligner()** - Constructor for class gata.aligner.DelmeAligner: **DelmeTest** - Class in cgar: **DelmeTest()** - Constructor for class cgar.DelmeTest: **demoUserId** - Static variable in class bioroot.Util: **DESCRIPTION\_TAG** - Static variable in class edu.utah.seq.parsers.BarParser: **DestypeIntensityCalculator** - Class in trans.qc: **DestypeIntensityCalculator()** - Constructor for class trans.qc.DestypeIntensityCalculator: **dH** - Static variable in class util.bio.calc.NucleicAcid: delta H values for dinucleotide pairs. **difference(float[][], float[][])** - Static method in class util.gen.Num: Returns the difference of each pair of average, aveT - aveC. **difference(int[], int[])** - Static method in class util.gen.Num: Returns the difference of each pair. **difference(double[], double[])** - Static method in class util.gen.Num: Returns the difference of each pair. **displayClusterPlot()** - Method in class util.bio.cluster.HierarchicalClustering: **divide(HashMap<String, PointData>, int)** - Static method in class edu.utah.seq.data.PointData: Randomly divides data. **DividePointData** - Class in edu.utah.seq.data: Merges Point Data and then divides using random sampling. **DividePointData()** - Constructor for class edu.utah.seq.data.DividePointData: **DmelCGNameCoordinatePrinter** - Class in trans.anno: Prints genome coordinates for Dmel Rel 4 GFF3 CG names. **DmelCGNameCoordinatePrinter(String[])** - Constructor for class trans.anno.DmelCGNameCoordinatePrinter: **DmelRel4Extractor** - Class in util.bio.parsers.gff: Class for building gene models from GFF3Features, specifically tailored to release 4.0 drosophila melanogaster GFF. **DmelRel4Extractor()** - Constructor for class util.bio.parsers.gff.DmelRel4Extractor: **DNAStat(String, String, int, int, int, int)** - Static method in class gata.main.GATAUtil: **doGet(HttpServletRequest, HttpServletResponse)** - Method in class bioroot.AccountManagement: **doGet(HttpServletRequest, HttpServletResponse)** - Method in class bioroot.antibody.AntibodyBase: **doGet(HttpServletRequest, HttpServletResponse)** - Method in class bioroot.antibody.AntibodyModify: **doGet(HttpServletRequest, HttpServletResponse)** - Method in class bioroot.antibody.AntibodySpreadSheet: **doGet(HttpServletRequest, HttpServletResponse)** - Method in class bioroot.antibody.AntibodyViewPreferences: **doGet(HttpServletRequest, HttpServletResponse)** - Method in class bioroot.antibody.NewAntibody: **doGet(HttpServletRequest, HttpServletResponse)** - Method in class bioroot.Archive: **doGet(HttpServletRequest, HttpServletResponse)** - Method in class bioroot.CellType: **doGet(HttpServletRequest, HttpServletResponse)** - Method in class bioroot.Contact: **doGet(HttpServletRequest, HttpServletResponse)** - Method in class bioroot.Gene: **doGet(HttpServletRequest, HttpServletResponse)** - Method in class bioroot.Login: **doGet(HttpServletRequest, HttpServletResponse)** - Method in class bioroot.Logout: **doGet(HttpServletRequest, HttpServletResponse)** - Method in class bioroot.Marker: **doGet(HttpServletRequest, HttpServletResponse)** - Method in class bioroot.oligo.NewOligo: **doGet(HttpServletRequest, HttpServletResponse)** - Method in class bioroot.oligo.OligoBase: **doGet(HttpServletRequest, HttpServletResponse)** - Method in class bioroot.oligo.OligoModify: **doGet(HttpServletRequest, HttpServletResponse)** - Method in class bioroot.oligo.OligoSpreadSheet: **doGet(HttpServletRequest, HttpServletResponse)** - Method in class bioroot.oligo.OligoUpload: **doGet(HttpServletRequest, HttpServletResponse)** - Method in class bioroot.oligo.OligoViewPreferences: **doGet(HttpServletRequest, HttpServletResponse)** - Method in class bioroot.Organism: **doGet(HttpServletRequest, HttpServletResponse)** - Method in class bioroot.plasmid.NewPlasmid: **doGet(HttpServletRequest, HttpServletResponse)** - Method in class bioroot.plasmid.PlasmidBase: **doGet(HttpServletRequest, HttpServletResponse)** - Method in class bioroot.plasmid.PlasmidModify: **doGet(HttpServletRequest, HttpServletResponse)** - Method in class bioroot.plasmid.PlasmidSpreadSheet: **doGet(HttpServletRequest, HttpServletResponse)** - Method in class bioroot.plasmid.PlasmidUpload: **doGet(HttpServletRequest, HttpServletResponse)** - Method in class bioroot.plasmid.PlasmidViewPreferences: **doGet(HttpServletRequest, HttpServletResponse)** - Method in class bioroot.Search: **doGet(HttpServletRequest, HttpServletResponse)** - Method in class bioroot.strain.NewStrain: **doGet(HttpServletRequest, HttpServletResponse)** - Method in class bioroot.strain.StrainBase: **doGet(HttpServletRequest, HttpServletResponse)** - Method in class bioroot.strain.StrainModify: **doGet(HttpServletRequest, HttpServletResponse)** - Method in class bioroot.strain.StrainSpreadSheet: **doGet(HttpServletRequest, HttpServletResponse)** - Method in class bioroot.strain.StrainUpload: **doGet(HttpServletRequest, HttpServletResponse)** - Method in class bioroot.strain.StrainViewPreferences: **doGet(HttpServletRequest, HttpServletResponse)** - Method in class bioroot.UserRegistration: **doGet(HttpServletRequest, HttpServletResponse)** - Method in class cgar.Login: **doGet(HttpServletRequest, HttpServletResponse)** - Method in class cgar.TestInBioTools: **doPost(HttpServletRequest, HttpServletResponse)** - Method in class bioroot.AccountManagement: **doPost(HttpServletRequest, HttpServletResponse)** - Method in class bioroot.antibody.AntibodyBase: **doPost(HttpServletRequest, HttpServletResponse)** - Method in class bioroot.antibody.AntibodyModify: **doPost(HttpServletRequest, HttpServletResponse)** - Method in class bioroot.antibody.AntibodySpreadSheet: **doPost(HttpServletRequest, HttpServletResponse)** - Method in class bioroot.antibody.AntibodyViewPreferences: **doPost(HttpServletRequest, HttpServletResponse)** - Method in class bioroot.antibody.NewAntibody: **doPost(HttpServletRequest, HttpServletResponse)** - Method in class bioroot.Archive: **doPost(HttpServletRequest, HttpServletResponse)** - Method in class bioroot.CellType: **doPost(HttpServletRequest, HttpServletResponse)** - Method in class bioroot.Contact: **doPost(HttpServletRequest, HttpServletResponse)** - Method in class bioroot.Gene: **doPost(HttpServletRequest, HttpServletResponse)** - Method in class bioroot.Login: **doPost(HttpServletRequest, HttpServletResponse)** - Method in class bioroot.Marker: **doPost(HttpServletRequest, HttpServletResponse)** - Method in class bioroot.oligo.NewOligo: **doPost(HttpServletRequest, HttpServletResponse)** - Method in class bioroot.oligo.OligoBase: **doPost(HttpServletRequest, HttpServletResponse)** - Method in class bioroot.oligo.OligoModify: **doPost(HttpServletRequest, HttpServletResponse)** - Method in class bioroot.oligo.OligoSpreadSheet: **doPost(HttpServletRequest, HttpServletResponse)** - Method in class bioroot.oligo.OligoUpload: **doPost(HttpServletRequest, HttpServletResponse)** - Method in class bioroot.oligo.OligoViewPreferences: **doPost(HttpServletRequest, HttpServletResponse)** - Method in class bioroot.Organism: **doPost(HttpServletRequest, HttpServletResponse)** - Method in class bioroot.plasmid.NewPlasmid: **doPost(HttpServletRequest, HttpServletResponse)** - Method in class bioroot.plasmid.PlasmidBase: **doPost(HttpServletRequest, HttpServletResponse)** - Method in class bioroot.plasmid.PlasmidModify: **doPost(HttpServletRequest, HttpServletResponse)** - Method in class bioroot.plasmid.PlasmidSpreadSheet: **doPost(HttpServletRequest, HttpServletResponse)** - Method in class bioroot.plasmid.PlasmidUpload: **doPost(HttpServletRequest, HttpServletResponse)** - Method in class bioroot.plasmid.PlasmidViewPreferences: **doPost(HttpServletRequest, HttpServletResponse)** - Method in class bioroot.Search: **doPost(HttpServletRequest, HttpServletResponse)** - Method in class bioroot.strain.NewStrain: **doPost(HttpServletRequest, HttpServletResponse)** - Method in class bioroot.strain.StrainBase: **doPost(HttpServletRequest, HttpServletResponse)** - Method in class bioroot.strain.StrainModify: **doPost(HttpServletRequest, HttpServletResponse)** - Method in class bioroot.strain.StrainSpreadSheet: **doPost(HttpServletRequest, HttpServletResponse)** - Method in class bioroot.strain.StrainUpload: **doPost(HttpServletRequest, HttpServletResponse)** - Method in class bioroot.strain.StrainViewPreferences: **doPost(HttpServletRequest, HttpServletResponse)** - Method in class bioroot.UserRegistration: **doPost(HttpServletRequest, HttpServletResponse)** - Method in class cgar.Login: **doubleArrayToString(double[], String)** - Static method in class util.gen.Misc: Returns a String separated by separator for each double[i]. **doubleArrayToString(double[], int, String)** - Static method in class util.gen.Num: Converts an array of double to a String with a defined number of decimal places and a delimiter. **doubleArrayToStringOnlyMax(double[], int, String)** - Static method in class util.gen.Num: Converts an array of double to a String with a maximal number of defined decimal places and a delimiter. **download(File, String[], String[])** - Method in class util.amazon.S3Manager: Downloads the keyed objects into the directory as files. **download(String, File, String[], String[])** - Method in class util.amazon.S3Manager: Downloads files from S3 using a different thread. **download(File)** - Method in class util.amazon.S3Manager: Uses the file name as the S3 key to fetch and save the file. **download(File[])** - Method in class util.amazon.S3Manager: Uses the file names as the S3 keys to fetch and save the files. **draw(double)** - Method in class gata.geneGlyphs.GeneRepGlyph: **draw(float, float, float, float, float, ArrayList, float, float, boolean)** - Method in class gata.geneGlyphs.GenericGlyph: **draw(double)** - Method in class gata.geneGlyphs.TransGrpGlyph: **draw(Graphics2D)** - Method in class trans.graphics.Box: **draw(Graphics2D, FontRenderContext, Font)** - Method in class trans.graphics.GenomicRegionGlyph: **draw(Graphics2D, FontRenderContext, Font)** - Method in class trans.graphics.Graph: **drawAlignment(Graphics2D)** - Method in class gata.aligner.Alignment: Called every time the alignPanel is repaint() ed **drawDryRunAnnotation()** - Method in class gata.geneGlyphs.Annotation: **drawGenericGlyphs()** - Method in class gata.geneGlyphs.Annotation: Call each time parameters effecting GenericGlyphs change **drawGlyphPanel(Graphics2D)** - Method in class gata.geneGlyphs.GlyphPanel: **drawGlyphs()** - Method in class gata.geneGlyphs.Annotation: Call each time the scale changes or user alters a glyph viewablility **drawJLabel(Graphics2D, JLabel, float)** - Static method in class util.gen.Swing: Draws a JLabel on a Graphics2D. **drawLabels(ArrayList, Font, Graphics2D)** - Static method in class gata.main.GATAUtil: **drawLines(Line2D.Double[], Graphics2D)** - Static method in class gata.main.GATAUtil: draws Shapes **drawTransGrpGlyphs()** - Method in class gata.geneGlyphs.GeneRepGlyph: **drop(int)** - Method in class edu.utah.seq.data.PointData: Randomly removes the numToDrop from the position and score arrays. **DrosGeneCollFileParser** - Class in util.bio.parsers: Parses and extracts a unique set of gene names from a drosophila gene collection file. **DrosGeneCollFileParser()** - Constructor for class util.bio.parsers.DrosGeneCollFileParser: **dS** - Static variable in class util.bio.calc.NucleicAcid: delta S values for dinucleotide pairs.

---


|  |  |  |
| --- | --- | --- |
| **Overview** \| Package \| Class \| Use \| **Tree** \| **Deprecated** \| **Index** \| **Help** | |  |
| **PREV LETTER**   **NEXT LETTER** | **FRAMES**    **NO FRAMES**     **All Classes** |  |


A B C D E F G H I J L M N O P Q R S T U V W X Z 

---
